# Leader-Follower Formation Control of UUVs with Model Uncertainties, Current Disturbances, and Unstable Communication

**DOI:** 10.3390/s18020662

**Published:** 2018-02-23

**Authors:** Zheping Yan, Da Xu, Tao Chen, Wei Zhang, Yibo Liu

**Affiliations:** College of Automation, Harbin Engineering University, Harbin 150001, China; yanzheping@hrbeu.edu.cn (Z.Y.); xuda@hrbeu.edu.cn (D.X.); dawizw@163.com (W.Z.); hnhbct@163.com (Y.L.)

**Keywords:** multi-UUV sensor networks, leader-follower formation control, model uncertainties, current disturbances, unstable communication

## Abstract

Unmanned underwater vehicles (UUVs) have rapidly developed as mobile sensor networks recently in the investigation, survey, and exploration of the underwater environment. The goal of this paper is to develop a practical and efficient formation control method to improve work efficiency of multi-UUV sensor networks. Distributed leader-follower formation controllers are designed based on a state feedback and consensus algorithm. Considering that each vehicle is subject to model uncertainties and current disturbances, a second-order integral UUV model with a nonlinear function is established using the state feedback linearized method under current disturbances. For unstable communication among UUVs, communication failure and acoustic link noise interference are considered. Two-layer random switching communication topologies are proposed to solve the problem of communication failure. For acoustic link noise interference, accurate representation of valid communication information and noise stripping when designing controllers is necessary. Effective communication topology weights are designed to represent the validity of communication information interfered by noise. Utilizing state feedback and noise stripping, sufficient conditions for design formation controllers are proposed to ensure UUV formation achieves consensus under model uncertainties, current disturbances, and unstable communication. The stability of formation controllers is proven by the Lyapunov-Razumikhin theorem, and the validity is verified by simulation results.

## 1. Introduction

In recent years, underwater mobile sensor networks have been rapidly developed and widely used in marine science and engineering fields. Compared with traditional static sensor networks, the underwater mobile sensor networks [1] can realize dynamic, large-scale sensing and operation at a lower cost. Due to the autonomous properties [2], multi-UUV, regarded as intelligent and reconfigurable underwater mobile sensor networks [3,4], have found an increasingly wide utilization for combined investigation, cooperative survey, and coordinated exploration [5]. Usually, multi-UUV sensor networks adopt a formation mode when sailing and working, which are propitious to information interaction and cooperative operation between UUVs. Thus, good formation control [6] is necessary and important for improving operation efficiency and reducing energy consumption of multi-UUVs.

There are four main methods for formation control [7], such as behavioral, virtual structure, queues and artificial potential trenches, and leader–follower approaches. For this paper, the leader–follower approach is adopted to realize formation control of multi-UUVs. In recent years, a great deal of attention has been focused on leader–follower formation control to keep multi-UUVs in a desired formation configuration and, at the same time, to complete the assigned tasks. Edwards [8] proposed a method that the leader navigates the mission waypoints and each follower maintains its place in formation using the position of the leader via an exogenous system with knowledge of the internal positions of all UUVs. In [9], the follower controller is designed by back-stepping and an approximate-based control method to track a reference trajectory based on the leader position and predetermined formation without the need for the leader’s velocity and dynamics. Bikramaditya [10] addresses the leader–follower formation control of multiple non-holonomic UUVs for the area coverage problem, based on a planned path by an optimization algorithm for the formation leader motion, and a designed communication strategy so that the UUVs can exchange information to obtain the designated waypoints that are sent from the leader. Therefore, one significant advantage of the leader–follower approach is that the reference trajectory is clearly defined by the leader and the internal formation stability is induced by the control laws of individual vehicles.

In general, the objective of coordinated formation control is to seek collaborative policies such that each UUV uses only limited local information to reach an overall goal in the ocean investigation, survey and exploration mission. This means that controllers of the leader-follower UUV formation should be designed to achieve consensus in complicated environments [11]. In recent years, considerable research efforts have been made on consensus [12,13,14]. A sufficient condition was derived to achieve multi-agent systems’ consensus in the case where the network is jointly connected frequently enough as the network evolves with time [15]. Second-order consensus in a multi-agent dynamical system with sampled data was studied by proposing a necessary and sufficient condition in [16,17]. Finite-time position consensus and collision avoidance problems are investigated for multi-UUV systems, which guarantee collision avoidance, connectivity maintenance, velocity matching, and consensus boundedness [18]. In fact, the particular underwater environment poses great disturbances on the formation control of multiple UUV system [19,20]. The controllers for multi-UUV should be designed for safety and robustness, especially in the presence of large model uncertainty and ocean current disturbances [21,22].

As is well known, in the difficult underwater communication environment, most communication systems on land cannot be applied in the design of UUVs, and the exchange of information between UUVs can only pass through limited underwater acoustic links which is characterized by low stability. In fact, how to design the formation control scheme while taking into account the practical means of communication for a multi-UUV system is a great challenge and has not been fully studied. In recent years, there are many studies regarding the narrow band and time delay of the acoustic communication of multiple UUVs [23,24], but fewer studies regarding the communication failures which have a serious impact on the stability of communications. Due to the link failure, which is a temporary disruption in an acoustic link caused by the complex underwater environment, the communication topology of multi-UUVs may fail to remain constant, but dynamically change over time. Moreover, the underwater acoustic links are seriously influenced by marine background noises. Thus, how to obtain valid communication information is very important for the UUV formation control.

Motivated by the above discussion, a formation control algorithm which can make the multiple UUV system achieve consensus is developed. The main contributions of this paper can be summarized as follows: First of all, a leader-follower UUV formation control approach is designed based on state feedback and consensus algorithm. To design the controller, the coupled mathematical model of the UUV is simplified and rewritten into a linearization model by state feedback linearization. Considering model uncertainties and current disturbances, a second-order integral UUV model with nonlinear function and current disturbances is established. Secondly, for unstable communication among UUVs, communication failure and acoustic link noise interference are considered. Two-layer random switching topologies, which are used to deliver position and velocity information, respectively, are adopted to improve the efficiency of communication and solve the problem of communication failure. For the acoustic link noise interference, the concept of communication topology effective weight is used to represent the validity of communication information interfered by noise in the acoustic link, and obtain the effective weights of position and velocity topologies, respectively. Finally, by stripping noise disturbances, sufficient conditions to design distributed controllers are proposed to ensure the UUV formation can achieve consensus under model uncertainties, current disturbances, and unstable communication. The stability of the leader-follower formation control is proven by using the Lyapunov-Razumikhin theorem.

The rest of this paper is organized as follows: Section 2 is the problem statement. In Section 3, the second-order integral UUV model with nonlinear function and current disturbances is built by state feedback. In Section 4, the leader-follower UUV formation controllers are designed by the state feedback and consensus algorithm under unstable communication. Section 5 presents the simulation results. Section 6 is the discussion of those results. Finally, Section 7 offers the conclusion.

## 2. Problem Statement 

### 2.1. Multi-UUV Sensor Networks

**Figure 1:**
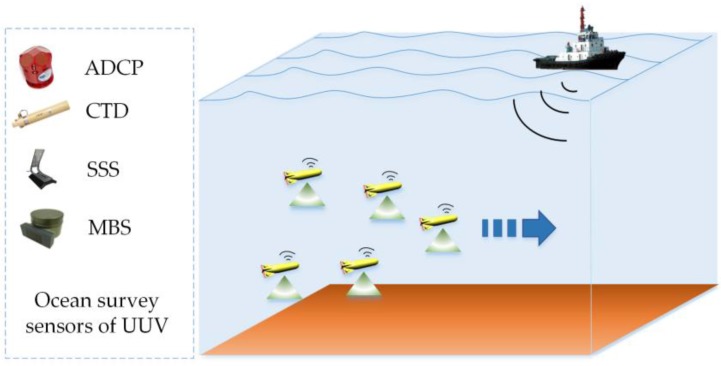
The multi-UUV sensor network.

The novel key feature of underwater sensor networks are multi-UUV sensor networks. Let UUV act as an intelligent sensing and operating node, and then the reconfigurable underwater mobile sensor networks are built for ocean investigation, survey, and exploration missions (Figure 1). In this paper, each UUV is equipped with one or more ocean survey sensors. The ocean survey sensors include: (1) an upward-looking ADCP (Acoustic Doppler Current Profiler) with a maximum range of 100 m; (2) a CTD (Conductivity Temperature Depth) sensor can continuously measure water conductivity, temperature, and depth; (3) side-scan sonar (SSS) creates an image of the sea floor topography for searching and detecting objects. The sonar is a dual frequency type that projects acoustic waves at 120 and 410 KHz at the central frequency; and (4) multi-beam echo sounder (MBS) observes bathymetry for mapping the seafloor terrain. Usually, multi-UUV sensor networks adopt a formation mode when sailing and working. Aiming to improve the work efficiency and reduce the energy consumption of multi-UUV sensor networks, the mission planning, navigation and location, and control of multi-UUVs are necessary. This paper mainly researches the leader-follower formation control method so that these UUVs can form an intelligent network achieving high performance with significant features of scalability, robustness, and reliability. The leader tracks a reference trajectory, and UUVs keep in a formation which is designed for specific tasks and mission areas.

The application area of the method and algorithm developed in this paper is mainly for ocean investigation, survey, and exploration missions using a middle-number-scale multi-UUV sensor network. Here, the middle-number-scale means that the multi-UUV system has no more than 10 UUVs, and, in order to realize formation sailing and operation, the multi-UUV system must have acoustic networking communication capabilities with a frequency range of 8–16 kHz. In addition, for obtaining better communication interaction and collaborative control, the formation spacing between every two UUVs is limited to 30–100 m.

### 2.2. Model Uncertainties, Current Disturbances, and Unstable Communication

**Figure 2:**
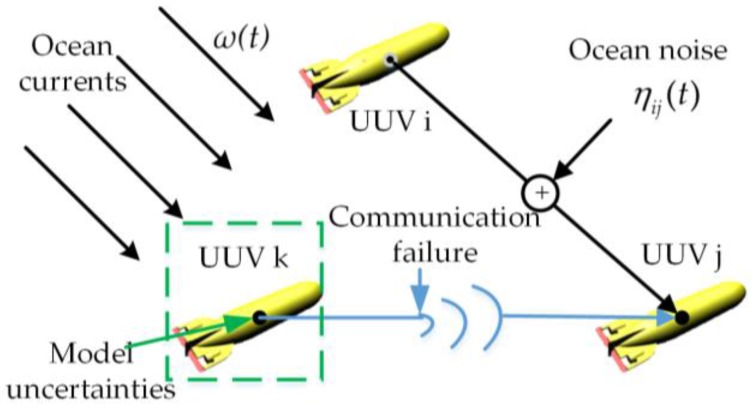
Model uncertainties, current disturbances, and unstable communication for UUV formation.

The particular underwater environment poses great influences on the stability of leader-follower UUV formation control, which is shown in Figure 2. The distributed formation controllers are designed based on state feedback and consensus algorithm in this paper. To design the controller, the coupled mathematical model of the UUV should be simplified into a second-order integral model by state feedback linearization. However, each vehicle is subject to model uncertainties and current disturbances, as the underwater movement of UUV is a complex space motion. The model uncertainties include time-varying parameters and additional nonlinear parts. Thus, the model of the UUV is divided into an approximate linear part and a nonlinear uncertain part in Section 3. A second-order integral UUV model with nonlinear function and current disturbances is established.

The state information between UUVs is transmitted through underwater acoustic links. There are many factors affecting the acoustic communication, and this paper mainly researches communication failures and acoustic link noise interference. The communication failure, data not available from the collaborative UUVs during certain periods of time, seriously limits information exchanges. Two-layer random switching topologies are adopted to solve the problem of communication failure. The topologies can dynamically change to maintain formation communication. In addition, the acoustic communication information may be disturbed by noises in the process from sender to receiver. How to accurately represent valid communication information and strip noise out of state information is researched.

### 2.3. Graph Theory

The communication relationship of all UUVs is G(V,ε,A), where V={1,2,⋯,n} is *n* UUVs nodes set, ε⊆V×V is edge set representing all communication links in formation, $A\in {\mathbb{R}}^{n\times n}$ is the adjacency matrix. The neighbor set of node i is denoted as $N_{i}=\{j\in V|(j,i)\in \varepsilon \}$. The Laplacian matrix $L=[l_{ij}]\in {\mathbb{R}}^{n\times n}$ associated with A is defined as L=D−A. $D=diag\{d_{1},d_{2},\ldots ,d_{n}\}$ is defined as the in-degree matrix.

The communication topologies of the whole UUV formation include two parts. One communication topology is among n followers, the other is between the leader and all followers. Considering the limitation of acoustic link bandwidth, communication links among all UUV members are divided into position information links and velocity information links. Thus, the communication topologies are divided into position topology ${\bar{G}}_{p}({\bar{V}}_{p},{\bar{\varepsilon }}_{p},{\bar{A}}_{p})$ and velocity topology ${\bar{G}}_{v}({\bar{V}}_{v},{\bar{\varepsilon }}_{v},{\bar{A}}_{v})$.

Let $G_{p}(V_{p},\varepsilon_{p},A_{p})$ and $G_{v}(V_{v},\varepsilon_{v},A_{v})$ be the position and velocity topologies among n followers. In ${\bar{G}}_{p}({\bar{V}}_{p},{\bar{\varepsilon }}_{p},{\bar{A}}_{p})$, make ${\bar{V}}_{p}=V_{p}\cup V_{l}$, ${\bar{\varepsilon }}_{p}=\varepsilon_{p}\cup \varepsilon_{l}$, where $V_{l}$ denotes the leader node, $\varepsilon_{l}$ is the communication link between the leader and the followers. ${\bar{G}}_{v}({\bar{V}}_{v},{\bar{\varepsilon }}_{v},{\bar{A}}_{v})$ is the same definition. The Laplacian matrix of ${\bar{G}}_{p}({\bar{V}}_{p},{\bar{\varepsilon }}_{p},{\bar{A}}_{p})$ is $L_{p}+L_{c}$, and the Laplacian matrix of ${\bar{G}}_{v}({\bar{V}}_{v},{\bar{\varepsilon }}_{v},{\bar{A}}_{v})$ is $L_{v}+L_{d}$. $L_{c}$ is the position adjacency matrix between the leader and the followers, and $L_{d}$ is the velocity adjacency matrix.

### 2.4. Consensus of the Leader-Follower UUV Formation

**Figure 3:**
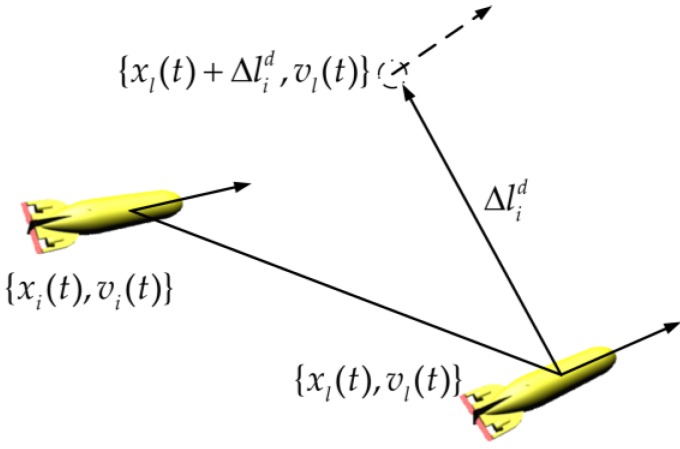
The leader-follower UUV formation.

In this paper, the leader is fully-functional, guides the whole formation, and can transmit its position and orientation $x_{l}(t)$, and velocity $v_{l}(t)$, to all followers. Each follower maintains a desired geometric configuration with the leader. For UUV leader-follower formation, the leader is defined as the fixed reference point $\left\{x_{l}(t),v_{l}(t)\right\}$. Thus, the expected state of each follower UUV is that $\left\{x_{l}(t)+\Delta l_{i}^{d},v_{l}(t)\right\}$, $\Delta l_{i}^{d}$ is the fixed relative distance to the leader (Figure 3).

The advantage of leader-follower formation is that specifying a single quantity (the leader’s motion) directs the group behavior. Therefore, it is simple since a reference trajectory is clearly defined by the leader and the internal formation stability is induced by the control laws of the followers. In this way, the conclusion is that each follower UUV can also converge to the desired point, if each follower UUV can converge to the leader in the formation. Thus, Definition 1 can be obtained as follows.

**Definition** **1.** *In the leader-follower UUV formation, there is one leader and n followers and, at time t, the motion state vector of the ith follower UUV is* $\varepsilon_{i}(t)={\left[\begin{array}{cc} x_{i}^{T}(t) & v_{i}^{T}(t) \end{array}\right]}^{T}$*, and the* *motion state vector of the leader is* $\varepsilon_{l}(t)={\left[\begin{array}{cc} x_{l}^{T}(t) & v_{l}^{T}(t) \end{array}\right]}^{T}$*. If the following formula holds, the leader-follower UUV formation can achieve consistency and continuously ensure the system stability and convergence:*

(1) $$\underset{t\to \infty }{\lim}\left|\varepsilon_{i}(t)-\varepsilon_{l}(t)\right|=0.$$

## 3. Second-Order Integral UUV Model with Nonlinear Function and Current Disturbances

### 3.1. Five-Degrees-of-Freedom UUV Model

The kinematics and dynamics model [25] of UUV is:

(2) $$\{\begin{array}{l} \dot{x}=J(x)v\\ M\dot{v}+C_{R}(v)v+Y(v)+g(x)=T \end{array}$$

where $x={\left[x,y,z,\theta ,\psi \right]}^{T}$ is the position and orientation vector, $v={\left[u,v,w,q,r\right]}^{T}$ is the linear velocity and angular velocity, $M=M_{R}+M_{A}$ includes the inertial matrix and additional inertial matrix, $C_{R}(v)$ denotes the Coriolis force, $g(x)={\left[0,0,0,z_{B}^{B}B\sin\theta ,0\right]}^{T}$ denotes the restoring force which is generated by the difference between the center of gravity and the center of buoyancy, $r_{B}^{W}=[0,0,0]$ is the coordinate of gravity center in the body-fixed frame, and $r_{B}^{B}=[x_{B}^{B},y_{B}^{B},z_{B}^{B}]$ is the coordinate of the buoyancy center in the body-fixed frame. $T={\left[X,Y,Z,M,N\right]}^{T}$ is the propulsion forces and moments vector. J(x) is the transformation between the body-fixed frame and the Earth reference frame. $Y(v)={\left[Y_{X},Y_{Y},Y_{Z},Y_{M},Y_{N}\right]}^{T}$ is the fluid viscosity force. There are:

$J(x)=\left[\begin{array}{cc} J_{1}(x) & 0\\ 0 & J_{2}(x) \end{array}\right]$, $J_{1}(x)=\left[\begin{array}{ccc} \cos\psi \cos\theta & -\sin\psi & \cos\psi \sin\theta \\ \sin\psi \cos\theta & \cos\psi & \sin\psi \sin\theta \\ -\sin\theta & 0 & \cos\theta \end{array}\right]$, $J_{2}(x)=diag\{1,1/\cos\theta \}$, and $C_{R}(v)=\left[\begin{array}{ccccc} 0 & -mr & mq & 0 & 0\\ mr & 0 & 0 & 0 & 0\\ -mq & 0 & 0 & 0 & 0\\ 0 & 0 & 0 & 0 & 0\\ 0 & 0 & 0 & 0 & 0 \end{array}\right]$.

The list of main symbols used in the paper is shown in Table A1 of the Appendix A.

### 3.2. State Feedback Linearization

Combine $C_{R}(v)v$, Y(v), and g(x) into a column vector N(x,v) which does not include inertia mass and additional inertial mass. The UUV Equation (2) is further rewritten as:

(3) $$\left[\begin{array}{c} \dot{x}\\ \dot{v} \end{array}\right]=\left[\begin{array}{cc} I & 0\\ 0 & -M^{-1} \end{array}\right]\left[\begin{array}{c} J(x)v\\ N(x,v) \end{array}\right]+\left[\begin{array}{c} 0\\ M^{-1} \end{array}\right]T,$$

where $N(x,v)=-(C_{R}(v)v+Y(v)+g(x))=\left[\begin{array}{l} mvr-mwq-Y_{X}\\ -mur-Y_{Y}\\ mvq-Y_{Z}\\ -z_{B}^{B}Bsin\theta -Y_{M}\\ -Y_{N} \end{array}\right]$.

In Equation (3), the two matrices consisting of $M^{-1}$ are:

(4) $$M_{1}=\left[\begin{array}{cc} I & 0\\ 0 & -M^{-1} \end{array}\right]\in {\mathbb{R}}^{10\times 10},M_{2}=\left[\begin{array}{c} 0\\ M^{-1} \end{array}\right]\in {\mathbb{R}}^{10\times 5},$$

where

(5) $$M^{-1}=\left[\begin{array}{ccccc} m_{11} & 0 & 0 & 0 & 0\\ 0 & m_{22} & 0 & 0 & m_{25}\\ 0 & 0 & m_{33} & m_{34} & 0\\ 0 & 0 & m_{43} & m_{44} & 0\\ 0 & m_{52} & 0 & 0 & m_{55} \end{array}\right].$$

To simplify the representation, let $\Sigma_{1}$ and $\Sigma_{2}$ be:

(6) $$\Sigma_{1}=(m-\frac{1}{2}\rho L^{3}Y_{\dot{v}}^{\prime })(I_{z}-\frac{1}{2}\rho L^{5}N_{\dot{r}}^{\prime })-(\frac{1}{2}\rho L^{4}Y_{\dot{r}}^{\prime })(\frac{1}{2}\rho L^{4}N_{\dot{v}}^{\prime }).$$

(7) $$\Sigma_{2}=(m-\frac{1}{2}\rho L^{3}Z_{\dot{w}}^{\prime })(I_{y}-\frac{1}{2}\rho L^{5}M_{\dot{q}}^{\prime })-(\frac{1}{2}\rho L^{4}Z_{\dot{q}}^{\prime })(\frac{1}{2}\rho L^{4}M_{\dot{w}}^{\prime }).$$

where $m_{11}=1/(m-\frac{1}{2}\rho L^{3}X_{\dot{u}}^{\prime })$, $m_{22}=(I_{z}-\rho L^{5}N_{\dot{r}}^{\prime }/2)/\Sigma_{1}$, $m_{25}=(-\rho L^{4}N_{\dot{v}}^{\prime }/2)/\Sigma_{1}$, $m_{33}=(m-\rho L^{3}Z_{\dot{w}}^{\prime }/2)/\Sigma_{2}$, $m_{34}=(-\rho L^{4}Z_{\dot{q}}^{\prime }/2)/\Sigma_{2}$, $m_{43}=(-\rho L^{4}M_{\dot{w}}^{\prime }/2)/\Sigma_{2}$, $m_{44}=(I_{y}-\rho L^{5}M_{\dot{q}}^{\prime }/2)/\Sigma_{2}$, $m_{52}=(-\rho L^{4}N_{\dot{v}}^{\prime }/2)/\Sigma_{1}$, $m_{55}=(m-\rho L^{3}Y_{\dot{v}}^{\prime }/2)/\Sigma_{1}$.

Then, the thrust $X_{prop},Y_{prop},Z_{prop}$ and steering angle $\delta_{r},\delta_{s}$ are control input $\hat{u}$, where $T=g^{\prime }(x)\hat{u}$:

(8) $$T=\left[\begin{array}{c} X\\ Y\\ Z\\ M\\ N \end{array}\right]=\left[\begin{array}{ccccc} 1 & 0 & 0 & 0 & 0\\ 0 & 1 & 0 & 0 & g_{24}^{\prime }\\ 0 & 0 & 1 & g_{34}^{\prime } & 0\\ 0 & 0 & 0 & g_{44}^{\prime } & 0\\ 0 & 0 & 0 & 0 & g_{55}^{\prime } \end{array}\right]\left[\begin{array}{c} X_{prop}\\ Y_{prop}\\ Z_{prop}\\ \delta_{s}\\ \delta_{r} \end{array}\right],$$

where $g_{44}^{\prime }=M_{\left|q\right|\delta_{s}}u\left|q\right|+M_{\delta_{s}}u^{2}$, $g_{34}^{\prime }=Z_{\delta_{s}}u\left|u\right|$, $g_{55}^{\prime }=N_{\left|r\right|\delta_{r}}u\left|r\right|+N_{\delta_{r}}u^{2}$, and $g_{24}^{\prime }=Y_{\delta_{r}}u\left|u\right|$.

The state vector $\varepsilon ={\left[x^{T},v^{T}\right]}^{T}$ is constituted by positions, orientations, and velocities of the UUV. Equation (3) can be rewritten as:

(9) $$\dot{\varepsilon }=f(\varepsilon )+M_{2}g^{\prime }(\varepsilon )\hat{u},$$

where $f(\varepsilon )=M_{1}\left[\begin{array}{c} J(x)v\\ N(x,v) \end{array}\right]\in {\mathbb{R}}^{10\times 1}$, $g^{\prime }(\varepsilon )=\left[g_{ij}^{\prime }(\varepsilon )\right]\in {\mathbb{R}}^{5\times 5}$. Let $g(\varepsilon )=M_{2}g^{\prime }(\varepsilon )$, define an output function y=h(ε), and obtain the general nonlinear model of the UUV:

(10) $$\left\{ \begin{array}{l} \dot{\varepsilon }=f(\varepsilon )+g(\varepsilon )\hat{u}\\ y=h(\varepsilon ) \end{array}\right..$$

Now, a feedback control law u through coordinate transformation [26] is designed to realize the feedback linearization of the UUV general nonlinear model. Considering Equation (10), let:

(11) $$h(\varepsilon )={\left[h_{1}(\varepsilon ),h_{2}(\varepsilon ),h_{3}(\varepsilon ),h_{4}(\varepsilon ),h_{5}(\varepsilon )\right]}^{T}={\left[x,y,z,\theta ,\psi \right]}^{T}.$$

According to the definition of Lie derivatives [27,28], Lie derivatives of general nonlinear model of the UUV are obtained:

(12) $$\dot{y}=\frac{\partial h}{\partial \varepsilon }\left[f(\varepsilon )+g(\varepsilon )u\right].$$

Then, the partial differential equation of h(ε) is:

(13) $$L_{f}h_{i}(\varepsilon )=f_{i}(\varepsilon ).$$

Since the first to the fifth elements of $g_{i}(\varepsilon )$ are zero, the equation $L_{g_{i}}h_{j}(\varepsilon )=0$ holds for any 1≤i≤5,1≤j≤5.

For notation simplicity, c⋅ and s⋅ represent cos⋅ and sin⋅ function respectively, then:

(14) $$\begin{array}{ll} L_{f}^{2}h_{1}(\varepsilon )= & \left(-uc\psi s\theta +wc\psi c\theta )f_{4}(\varepsilon )+(-us\psi c\theta -vc\psi -ws\psi s\theta )f_{5}(\varepsilon \right)\\ & \;+c\psi c\theta f_{6}(\varepsilon )-s\psi f_{7}(\varepsilon )+c\psi s\theta f_{8}(\varepsilon ), \end{array}$$

(15) $$\begin{array}{ll} L_{f}^{2}h_{2}(\varepsilon )= & \left(-us\psi s\theta +ws\psi c\theta )f_{4}(\varepsilon )+(uc\psi c\theta -vs\psi +wc\psi s\theta )f_{5}(\varepsilon \right)\\ & \;+s\psi c\theta f_{6}(\varepsilon )+c\psi f_{7}(\varepsilon )+s\psi s\theta f_{8}(\varepsilon ), \end{array}$$

(16) $$L_{f}^{2}h_{3}(\varepsilon )=(-uc\theta -ws\theta )f_{4}(\varepsilon )-s\theta f_{6}(\varepsilon )+\cos\theta f_{8}(\varepsilon ),$$

(17) $$L_{f}^{2}h_{4}(\varepsilon )=f_{9}(\varepsilon ),$$

(18) $$L_{f}^{2}h_{5}(\varepsilon )=\frac{r\sin\theta }{\cos^{2}\theta }f_{4}(\varepsilon )+\frac{f_{10}(\varepsilon )}{\cos\theta }.$$

The matrix $\Gamma (\varepsilon )=[\Gamma_{ij}(\varepsilon )]\in {\mathbb{R}}^{5\times 5}$ consists of $L_{g_{i}}L_{f}h_{j}(\varepsilon ),1\leq i\leq 5,1\leq j\leq 5$, where $\Gamma_{ij}(\varepsilon )=L_{g_{j}}L_{f}h_{i}(\varepsilon )$.

According to the definition of relative degree, $\rho_{1}+\rho_{2}+\rho_{3}+\rho_{4}+\rho_{5}=10$ means that the number of system degrees are the same as relative degrees. Subtracting the redundancy terms, the states of the UUV is adopted as system outputs. Thus, the coordinate transformation is chosen as z=φ(ε) which are shown as follows:

(19) $$\left\{ \begin{array}{l} z_{1}={\left[h_{1}(\varepsilon ),h_{2}(\varepsilon ),h_{3}(\varepsilon ),h_{4}(\varepsilon ),h_{5}(\varepsilon )\right]}^{T}\\ z_{2}={\left[L_{f}h_{1}(\varepsilon ),L_{f}h_{2}(\varepsilon ),L_{f}h_{3}(\varepsilon ),L_{f}h_{4}(\varepsilon ),L_{f}h_{5}(\varepsilon )\right]}^{T} \end{array}\right..$$

According to the definition of Lie derivatives, yields:

(20) $$\left\{ \begin{array}{l} {\dot{z}}_{1}=z_{2}\\ {\dot{z}}_{2}=L_{f}^{2}h(\varepsilon )+L_{g}L_{f}h(\varepsilon )\hat{u} \end{array}\right..$$

For the new coordinate system, the control input u is:

(21) $$u=B(\varepsilon )+\Gamma (\varepsilon )\hat{u}=L_{f}^{2}h(\varepsilon )+L_{g}L_{f}h(\varepsilon )\hat{u}.$$

The feedback control input is $\hat{u}=\Gamma^{-1}(\varepsilon )\left(u-B(\varepsilon )\right)$. As a result, the linear second order integral UUV model can be obtained:

(22) $$\left\{ \begin{array}{l} {\dot{z}}_{1}=z_{2}\\ {\dot{z}}_{2}=u \end{array}\right..$$

### 3.3. Current Disturbances

Ocean current is one of main external disturbances of UUV. Current is changing with several factors such as sea area, depth, time, hydrology, salinity, and so on. It is difficult to describe an ocean current with a specific function. Supposing that the ocean current velocities in the Earth reference frame is $U_{E}=(u_{e},v_{e},w_{e})$, ocean current velocities $U_{B}=(u_{b},v_{b},w_{b})$ in the body-fixed frame can be obtained based on conversion matrix $J_{1}(x)$ [29]:

(23) $$\left[\begin{array}{c} u_{b}\\ v_{b}\\ w_{b} \end{array}\right]=J_{1}^{-1}(x)\left[\begin{array}{c} u_{e}\\ v_{e}\\ w_{e} \end{array}\right].$$

Since the control system of the leader-follower UUV formation has been represented by feedback linearization, the relative velocities of the UUV subject to $U_{B}$ is given:

(24) $$\left[\begin{array}{l} u_{r}\\ v_{r}\\ w_{r} \end{array}\right]=\left[\begin{array}{l} u\\ v\\ w \end{array}\right]-\left[\begin{array}{l} u_{b}\\ v_{b}\\ w_{b} \end{array}\right].$$

Substituting Equation (24) into the processes of feedback linearization, ocean current velocities can be separated from the relative velocity after coordinate conversion. Then, the velocity states of UUV can be described as:

(25) $$\hat{v}(t)=v(t)+\omega (t).$$

Assuming that UUV formation’s mission area is limited, the current function ω(t) is continuously bounded, and $\omega (t)={\left[u_{e},v_{e},w_{e},0,0\right]}^{T}$.

### 3.4. Model Uncertainties

The model uncertainties of the UUV include time-varying parameters and additional nonlinear parts which affect the stability of the UUV control system. Thus, the model uncertainties of the UUV must be added into the linear model introduced in Section 3.2, defining a bounded nonlinear function $f(x_{i},v_{i})$, which denotes the time-varying parameters and nonlinear terms. Then, the model of the UUV is divided into an approximate linear part in Equation (22) and a nonlinear uncertain part $f(x_{i},v_{i})$. Additionally, an assumption of $f(x_{i},v_{i})$ is built as follows:

**Assumption** **1.** *The bounded nonlinear function* $f(x_{i},v_{i}):\mathbb{R}\times {\mathbb{R}}^{n}\to {\mathbb{R}}^{n}$ *satisfies the following inequality:*

(26) $$\left\|f(x_{i}(t),v_{i}(t))-f(x_{j}(t),v_{j}(t))\right\|\leq \beta \left\|\varepsilon_{i}(t)-\varepsilon_{j}(t)\right\|,$$

*where* $\varepsilon_{i}={\left[\begin{array}{cc} x_{i}^{T} & v_{i}^{T} \end{array}\right]}^{T}$*,* $f(x_{i}(t),v_{i}(t))={\left[\begin{array}{cc} 0 & f_{v}(x_{i}(t),v_{i}(t)) \end{array}\right]}^{T}$*, and* β *is a positive real number.*

Here, it is considered that the model uncertainties exist as a certain probability. Thus, Bernoulli's distribution function ϑ(t) is used to describe the existence of the additional nonlinear function $f(x_{i},v_{i})$:

(27) Pr(ϑ(t)=1)=p,

(28) Pr(ϑ(t)=0)=1−p,

where ϑ(t)=1 shows the presence of the additional nonlinear function $f(x_{i},v_{i})$, otherwise it does not exist. p indicates the probability of its existence. Then the following equation must be established:

(29) E{ϑ(t)−p}=0.

In the presence of the model uncertainties and current disturbances, the leader’s model is:

(30) $$\left\{ \begin{array}{l} {\dot{x}}_{l}(t)=v_{l}(t)+\omega_{l}(t)\\ {\dot{v}}_{l}(t)=\vartheta (t)f_{v}(x_{l}(t),v_{l}(t)) \end{array}\right.,$$

The *i*th follower’s model is:

(31) $$\left\{ \begin{array}{l} {\dot{x}}_{i}(t)=v_{i}(t)+\omega_{i}(t)\\ {\dot{v}}_{i}(t)=u_{i}(t)+\vartheta (t)f_{v}(x_{i}(t),v_{i}(t)) \end{array}\right..$$

## 4. Formation Control with Unstable Communication

### 4.1. Two-Layer Random Switching Topologies for Communication Failure

Two-layer random switching communication topologies including position topology and velocity topology are proposed to solve the problem of communication failure in this part. Driven by the Markov random process, the position topology switches randomly among a position topology set, and the velocity topology switches randomly among a velocity topology set. Assuming that a topology set is $G_{}^{u}=\left\{G_{1},G_{2},\ldots ,G_{N}\right\}$, define a basic probability space of the Markov random process θ(t) is (Ω,F,P). F is the algebra of events, and P is the probability measure defined on *F*. When θ(t)=i, it indicates that the current communication topology is $G_{i},i\in \{1,2,\ldots ,N\}$, N is the quantity of the topological set. According to [30], the switching probability matrix is $\Gamma =[\gamma_{ij}]\in {\mathbb{R}}^{N\times N}$, then:

(32) $$P\left(\theta (t+\Delta t)=j|\theta (t)=i\right)=\left\{ \begin{array}{l} \gamma_{ij}\Delta t+o(\Delta t)\\ 1+\gamma_{ii}\Delta t+o(\Delta t) \end{array}\right.\begin{array}{c} if\;i\neq j\\ if\;i=j \end{array},$$

where $\gamma_{ij}$ represents the switching probability from topology i to topology j, and Δt>0. When i=j, $\gamma_{ii}=-\sum_{i\neq j}\gamma_{ij}$. o(Δt) denotes an infinitesimal of a higher order than Δt, which means $\underset{t\to \infty }{\lim}[o(\Delta t)/\Delta t]=0$.

Now, the position topology set and the velocity topology set are formed, respectively. Let ${\bar{G}}_{pi}({\bar{V}}_{pi},{\bar{\varepsilon }}_{pi},{\bar{A}}_{pi})$ be the *i*th position topological unit in the position topology set, 1≤i≤N. Joint topology of the position topology set is:

(33) $${\bar{G}}_{p}^{u}≜{\bar{G}}_{p1}\cup \ldots \cup {\bar{G}}_{pN}≜\left\{{\bar{V}}_{p}^{u},{\bar{\varepsilon }}_{p}^{u},{\bar{A}}_{p}^{u}\right\},$$

where ${\bar{V}}_{p}^{u}={\bar{V}}_{p1}\cup \ldots \cup {\bar{V}}_{pN}$, ${\bar{\varepsilon }}_{p}^{u}={\bar{\varepsilon }}_{p1}\cup \ldots \cup {\bar{\varepsilon }}_{pN}$, and ${\bar{A}}_{p}^{u}=\sum_{i=1}^{N}{\bar{A}}_{i}$.

In the same way, the joint topology of velocity topology set is obtained as ${\bar{G}}_{v}^{u}≜{\bar{G}}_{v1}\cup \ldots \cup {\bar{G}}_{vN}≜\left\{{\bar{V}}_{v}^{u},{\bar{\varepsilon }}_{v}^{u},{\bar{A}}_{v}^{u}\right\}$. The joint Laplacian matrix is: $L_{p}^{u}+L_{c}^{u}=\overset{N}{\underset{i=1}{\cup }}L_{pi}+\overset{N}{\underset{i=1}{\cup }}L_{ci}$, $L_{v}^{u}+L_{d}^{u}=\overset{N}{\underset{i=1}{\cup }}L_{vi}+\overset{N}{\underset{i=1}{\cup }}L_{di}$.

### 4.2. The Effective Weight of Communication Topologies for Ocean Noises

The underwater acoustic links are seriously subject to ocean noises. The state information of the UUV may be disturbed by noises in the process from sender to receiver. Gaussian white noise $\eta_{ij}(t)$ is used to model ocean noises in this paper. Thus, the communication topology weights are stochastically perturbed by Gaussian white noise $\eta_{ij}(t)$. ${\tilde{a}}_{ij}(t)\in A_{p}$ and ${\tilde{b}}_{ij}(t)\in A_{v}$ are respectively defined as the real position topology and velocity topology weights, which are interfered by $\eta_{ij}(t)$ among all follower UUVs. ${\tilde{a}}_{ij}=1$, ${\tilde{b}}_{ij}=1$, indicates that node j can receive information from node i, otherwise ${\tilde{a}}_{ij}=0$, ${\tilde{b}}_{ij}=0$. ${\tilde{c}}_{li}(t)$ is defined as the real position communication topology weights between the leader and followers, and the real velocity communication topology weights ${\tilde{d}}_{li}(t)$. ${\tilde{c}}_{li}=1$, ${\tilde{d}}_{li}=1$, indicate that the *i*th follower can receive the position and velocity information of the leader, otherwise ${\tilde{c}}_{li}=0$, ${\tilde{d}}_{li}=0$.

In order to solve the influence of ocean noise on communication, the concept of communication topology effective weight is introduced. Here, $0\leq a_{ij}(t)\leq 1$ and $0\leq b_{ij}(t)\leq 1$ are defined as position and velocity communication topology effective weights among all follower UUVs. $0\leq c_{li}(t)\leq 1$ and $0\leq d_{li}(t)\leq 1$ are defined as the position and velocity communication topology effective weights between the leader and followers. With the increasing of influence of ocean noises on communication, the communication topology effective weights decrease. Then, the relationships between real weights ${\tilde{a}}_{ij}(t)$, ${\tilde{b}}_{ij}(t)$ and effective weights $a_{ij}(t)$, $b_{ij}(t)$ can be expressed as:

(34) $${\tilde{a}}_{ij}(t)=a_{ij}(t)+\delta_{ij}(t)\eta_{ij}(t)a_{ij}(t),$$

(35) $${\tilde{b}}_{ij}(t)=b_{ij}(t)+\delta_{ij}(t)\eta_{ij}(t)b_{ij}(t),$$

where $\delta_{ij}(t)$ is the noise density in the link from sender i to receiver j at time t, and it is also a continuous function that varies with time. $\eta_{ij}(t)$ is Gaussian white noise in the communication link from sender i to receiver j at time t, and $\left\{\eta_{ij}(t),1\leq i,j\leq N\right\}$ is an independent standard Gaussian white noise.

In a similar way, the relationships between real weights ${\tilde{c}}_{li}(t)$, ${\tilde{d}}_{li}(t)$ and effective weights $c_{li}(t)$, $d_{li}(t)$ are:

(36) $${\tilde{c}}_{li}(t)=c_{li}(t)+\delta_{li}(t)\eta_{li}(t)c_{li}(t),$$

(37) $${\tilde{d}}_{li}(t)=d_{li}(t)+\delta_{li}(t)\eta_{li}(t)d_{li}(t).$$

Now, the validity of communication information can be expressed by effective communication topology weights instead of real communication topology weights.

### 4.3. Leader-Follower UUV Formation Control

According to Definition 1 and UUV model with nonlinear function and current disturbances, the control inputs at time t of leader-follower UUV formation with unstable communication are:

(38) $$\begin{array}{ll} u_{i}(t)= & K_{p}\sum_{j\in N_{i}^{p}}{\tilde{a}}_{ij}(t)\left(x_{j}(t)-x_{i}(t-\tau )\right)+K_{v}\sum_{j\in N_{i}^{p}}{\tilde{b}}_{ij}(t)\left(v_{j}(t)-v_{i}(t-\tau )\right)\\ & +K_{p}{\tilde{c}}_{li}(t)\left(x_{l}(t-\tau )-x_{i}(t-\tau )\right)+K_{v}{\tilde{d}}_{li}(t)\left(v_{l}(t-\tau )-v_{i}(t-\tau )\right) \end{array},$$

where $K_{p}$ and $K_{v}$ respectively represent the control gains for the position and velocity communication topologies, $N_{i}^{p}$ is the set of the *i*th UUV’s neighbors in the position topology, $N_{i}^{v}$ is the set of the *i*th UUV’s neighbors in the velocity topology, and τ represents a time-varying time delay τ(t).

Stripping noise interference, the noise interference is expressed as corresponding vectors, then the *i*th UUV’s interferences are:

(39) $$\Delta_{pi}(t)=\sum_{j\in N_{i}^{p}}\delta_{ij}(t)\eta_{ij}(t)a_{ij}(t)\left(x_{j}(t-\tau )-x_{i}(t-\tau )\right)+\delta_{li}(t)\eta_{li}(t)c_{li}(t)\left(x_{l}(t-\tau )-x_{i}(t-\tau )\right),$$

(40) $$\Delta_{vi}(t)=\sum_{j\in N_{i}^{p}}\left(\delta_{ij}(t)\eta_{ij}(t)b_{ij}(t)\right)\left(v_{j}(t-\tau )-v_{i}(t-\tau )\right)+\delta_{li}(t)\eta_{li}(t)d_{li}(t)\left(v_{l}(t-\tau )-v_{i}(t-\tau )\right).$$

The *i*th follower’s model with white noise interference, nonlinear function, and current disturbances is:

(41) $$\begin{array}{ll} {\dot{x}}_{i}(t)= & v_{i}(t)+\omega_{i}(t)\\ {\dot{v}}_{i}(t)= & K_{p}\sum_{j\in N_{i}^{p}}a_{ij}(t)\left(x_{j}(t)-x_{i}(t-\tau )\right)+K_{v}\sum_{j\in N_{i}^{p}}b_{ij}(t)\left(v_{j}(t)-v_{i}(t-\tau )\right)\\ & +K_{p}c_{li}(t)\left(x_{l}(t-\tau )-x_{i}(t-\tau )\right)+K_{v}d_{li}(t)\left(v_{l}(t-\tau )-v_{i}(t-\tau )\right)\\ & +K_{p}\Delta_{pi}(t)+K_{v}\Delta_{vi}(t)+\vartheta (t)f_{v}(x_{i}(t),v_{i}(t)) \end{array},$$

Defining the *i*th follower’s state vector is $\varepsilon_{i}(t)={\left[\begin{array}{cc} x_{i}^{T}(t) & v_{i}^{T}(t) \end{array}\right]}^{T}$, the leader’s state vector is $\varepsilon_{l}(t)={\left[\begin{array}{cc} x_{l}^{T}(t) & v_{l}^{T}(t) \end{array}\right]}^{T}$. Then, the state vectors of all followers are $\varepsilon (t)={\left[\begin{array}{cccc} \varepsilon_{1}^{T}(t) & \varepsilon_{2}^{T}(t) & \cdots & \varepsilon_{n}^{T}(t) \end{array}\right]}^{T}$. The system state equation is:

(42) $$\begin{array}{ll} \dot{\varepsilon }(t)= & \left(I_{n}⊗A\right)\varepsilon (t)-\left(\left(L_{p}+L_{c}\right)⊗{\bar{K}}_{p}\right)\varepsilon (t-\tau )-\left(\left(L_{v}+L_{c}\right)⊗{\bar{K}}_{v}\right)\varepsilon (t-\tau )\\ & \;+\left(L_{c}⊗{\bar{K}}_{p}\right)\varepsilon_{l}(t-\tau )+\left(L_{d}⊗{\bar{K}}_{v}\right)\varepsilon_{l}(t-\tau )+\left(I_{n}⊗{\bar{K}}_{p}\right)\Delta (t-\tau )\\ & \;+\left(I_{n}⊗{\bar{K}}_{v}\right)\Delta (t-\tau )+\Xi F_{\varepsilon }(t)+W(t) \end{array},$$

where $F_{\varepsilon }(t)=[f^{T}(x_{1}(t),v_{1}(t)),f^{T}(x_{2}(t),v_{2}(t)),\ldots ,f^{T}(x_{n}(t),v_{n}(t))]$ donates the nonlinear function of the system, and $\Xi =\vartheta (t)\left(I_{n}⊗I_{10}\right)$. $W(t)={\left[w_{1}^{T}(t),w_{2}^{T}(t),\ldots ,w_{n}^{T}(t)\right]}^{T}$ is the current disturbances vector, and $w_{i}(t)={\left[\begin{array}{cc} \omega_{i}^{T}(t) & 0 \end{array}\right]}^{T}$; $\Delta (t)={\left[\Delta_{1}^{T}(t),\Delta_{2}^{T}(t),\ldots ,\Delta_{n}^{T}(t)\right]}^{T}$, $\Delta_{i}(t)={\left[\Delta_{pi}^{T}(t),\Delta_{vi}^{T}(t)\right]}^{T}$, and $A=\left[\begin{array}{cc} 0 & I\\ 0 & 0 \end{array}\right]$, ${\bar{K}}_{p}=\left[\begin{array}{cc} 0 & 0\\ K_{p} & 0 \end{array}\right]$, ${\bar{K}}_{v}=\left[\begin{array}{cc} 0 & 0\\ K_{v} & 0 \end{array}\right]$.

According to Definition 1, the system’s state error vector is defined as $\xi (t)={\left[\xi_{1}^{T}(t),\ldots ,\xi_{n}^{T}(t)\right]}^{T}$, and the *i*th follower’s state error vector is $\xi_{i}(t)=\varepsilon_{i}(t)-\varepsilon_{l}(t)$.

In order to analyze ocean noise interference on system stability, Gaussian white noise is taken as a state variable of the system, which is written as follows:

(43) $$\Delta_{pi}(t)=X_{pi}^{\theta (t)=k}\eta_{i}(t)+Y_{pi}^{\theta (t)=k}\eta_{li}(t),$$

(44) $$\Delta_{vi}(t)=X_{vi}^{\theta (t)=k}\eta_{i}(t)+Y_{vi}^{\theta (t)=k}\eta_{li}(t),$$

and:

$$X_{pi}^{\theta (t)=k}=e_{pi}(t)\left({\bar{\delta }}_{i}{\bar{a}}_{i}^{\theta (t)=k}(t)\right),Y_{pi}^{\theta (t)=k}=\delta_{li}(t)c_{li}(t)\xi_{pi}(t),X_{vi}^{\theta (t)=k}=e_{vi}(t)\left({\bar{\delta }}_{i}{\bar{a}}_{i}^{\theta (t)=k}(t)\right),Y_{vi}^{\theta (t)=k}=\delta_{li}(t)c_{li}(t)\xi_{vi}(t),$$

where $\eta_{i}(t)={\left[\eta_{i1}^{T}(t),\eta_{i2}^{T}(t),\ldots ,\eta_{in}^{T}(t)\right]}^{T}\in {\mathbb{R}}^{n\times 1}$, ${\bar{\delta }}_{i}(t)=diag\left\{\delta_{i1}(t),\delta_{i2}(t),\ldots ,\delta_{in}(t)\right\}\in {\mathbb{R}}^{n\times n}$, ${\bar{a}}_{i}^{\theta (t)=k}(t)=diag\{a_{1}^{\theta (t)=k}(t),a_{2}^{\theta (t)=k}(t),\ldots ,a_{n}^{\theta (t)=k}(t)\}\in {\mathbb{R}}^{n\times n}$, $e_{pi}(t)=\left[\left(\xi_{p1}(t)-\xi_{pi}(t)\right),\left(\xi_{p2}(t)-\xi_{pi}(t)\right),\ldots ,\left(\xi_{pn}(t)-\xi_{pi}(t)\right)\right]\in {\mathbb{R}}^{5\times n}$, $e_{vi}(t)=\left[\left(\xi_{v1}(t)-\xi_{vi}(t)\right),\left(\xi_{v2}(t)-\xi_{vi}(t)\right),\ldots ,\left(\xi_{vn}(t)-\xi_{vi}(t)\right)\right]\in {\mathbb{R}}^{5\times n}$.

In addition, θ(t)=k denotes the Markov random process, k∈N. $\xi_{pi}(t)=x_{i}(t)-x_{l}(t)$, $\xi_{vi}(t)=v_{i}(t)-v_{l}(t)$. Assume that: $\eta (t)={\left[\eta_{1}^{T}(t),\eta_{2}^{T}(t),\ldots ,\eta_{n}^{T}(t),\eta_{1}^{T}(t),\eta_{2}^{T}(t),\ldots ,\eta_{n}^{T}(t)\right]}^{T}$, and $\eta_{l}(t)={\left[\eta_{l1},\ldots ,\eta_{ln},\eta_{l1},\ldots ,\eta_{ln}\right]}^{T}$. Obviously, the following matrices are available:

(45) $$X_{}^{\theta (t)=k}=diag\left\{X_{p}^{\theta (t)=k},X_{v}^{\theta (t)=k}\right\}\in {\mathbb{R}}^{10n^{2}\times 2n^{2}},$$

(46) $$Y_{}^{\theta (t)=k}=diag\left\{Y_{p}^{\theta (t)=k},Y_{v}^{\theta (t)=k}\right\}\in {\mathbb{R}}^{10n^{2}\times 2n^{2}},$$

(47) $$\Delta (t)=X_{}^{\theta (t)=k}\eta (t)+Y_{}^{\theta (t)=k}\eta_{l}(t),$$

where $X_{p}^{\theta (t)=k}=diag\left\{X_{p1}^{\theta (t)=k},X_{p2}^{\theta (t)=k},\ldots ,X_{pn}^{\theta (t)=k}\right\}$, $Y_{p}^{\theta (t)=k}=diag\left\{Y_{pi}^{\theta (t)=k},Y_{p2}^{\theta (t)=k},\ldots ,Y_{pn}^{\theta (t)=k}\right\}$,

$X_{v}^{\theta (t)=k}=diag\left\{X_{v1}^{\theta (t)=k},X_{v2}^{\theta (t)=k},\ldots ,X_{vn}^{\theta (t)=k}\right\}$, $Y_{v}^{\theta (t)=k}=diag\left\{Y_{vi}^{\theta (t)=k},Y_{v2}^{\theta (t)=k},\ldots ,Y_{vn}^{\theta (t)=k}\right\}$.

The system error state equation is obtained:

(48) $$\begin{array}{ll} \dot{\xi }(t)= & \left(I_{n}⊗A\right)\xi (t)-\left(\left(L_{p}+L_{c}\right)⊗{\bar{K}}_{p}\right)\xi (t-\tau )+\left(I_{n}⊗{\bar{K}}_{p}\right)\Delta (t-\tau )\\ & -\left(\left(L_{v}+L_{c}\right)⊗{\bar{K}}_{v}\right)\xi (t-\tau )+\left(I_{n}⊗{\bar{K}}_{v}\right)\Delta (t-\tau )+\Xi F(t)+\hat{W}(t) \end{array},$$

where, $F(t)={\left[\Delta f_{1},\Delta f_{2},\ldots ,\Delta f_{n}\right]}^{T}$, $\Delta f_{i}=f^{T}(x_{i}(t),v_{i}(t))-f^{T}(x_{l}(t),v_{l}(t))$, $\hat{W}(t)$ represents the vector of the current error between the followers and leader, and $\hat{W}(t)=W(t)-1_{n}w_{l}(t)$, $w_{l}(t)={\left[\begin{array}{cc} \omega_{l}^{T} & 0 \end{array}\right]}^{T}$.

Further simplified, Equation (48) can be written as:

(49) $$\begin{array}{ll} \dot{\xi }(t)= & \;\bar{A}\;\xi (t)-H_{p}^{\theta (t)=k}\xi (t-\tau )+{\hat{K}}_{p}\Delta (t-\tau )-H_{v}^{\theta (t)=k}\xi (t-\tau )+{\hat{K}}_{v}\Delta (t-\tau )\\ & +\Xi F(t)+\hat{W}(t) \end{array},$$

where $H_{p}^{\theta (t)=k}=\left(L_{p}+L_{c}\right)⊗{\bar{K}}_{p}$, $H_{v}^{\theta (t)=k}=\left(L_{v}+L_{d}\right)⊗{\bar{K}}_{v}$, ${\hat{K}}_{p}=I_{n}⊗{\bar{K}}_{p}$, ${\hat{K}}_{v}=I_{n}⊗{\bar{K}}_{v}$.

According to the state error, as shown in Equation (49), the sufficient conditions of stable convergence of the system are obtained as shown in Theorem 1.

**Theorem** **1.** *In a leader-follower UUV formation consisting of* n+1 *UUVs, the communication topologies satisfy the two-layer Markov random process. If the acoustic link is disturbed by ocean noise, at least one follower can receive the leader’s state information. If the following matrix inequality holds and positive definite matrices* P*,* $Q_{1}$ *and* $Q_{2}$ *exist, the leader-follower UUV formation control is stable and convergent:*

(50) $$\left[\begin{array}{ccc} P\;\bar{A}+{\bar{A}}^{T}P+Q_{1}+\Omega +\Sigma_{1} & -P\left(H_{p}+H_{v}\right) & 0\\ {}* & -(1-\kappa )\left(Q_{1}-Q_{2}\right)+\Sigma_{2} & 0\\ {}* & * & -(1-\kappa )Q_{2} \end{array}\right]<0,$$

*where* $\Sigma_{1}=N\mu_{1}\mathrm{PP}+\mu_{2}N\mathrm{PP}+\mu_{3}\mathrm{PP}+\mu_{4}\mathrm{PP}$*,* $\Omega =\left(\mu_{3}^{-1}\beta p^{2}+\varpi^{2}\gamma^{2}\mu_{4}^{-1}\right)I$*,* 0<p,δ<1*,* $\Sigma_{2}=2\mu_{2}^{-1}\delta^{2}\left(\left(L_{v}^{u}+L_{d}^{u}\right)⊗{\bar{K}}_{p}^{T}{\bar{K}}_{p}\right)+2\mu_{1}^{-1}\delta^{2}\left(\left(L_{p}^{u}+L_{c}^{u}\right)⊗{\bar{K}}_{p}^{T}{\bar{K}}_{p}\right)$*,* $\mu_{1},\mu_{2},\mu_{3},\mu_{4}>0$*, and* β,ϖ,γ>0*.*

### 4.4. Stability Analysis

Using the Lyapunov-Krasovskii theory to verify the stability of the leader-follower UUV formation control in the presence of model uncertainties, current disturbances, and unstable communication, build the Lyapunov function:

(51) $$V(t)=\xi^{T}(t)P\xi (t)+\int_{t-\tau }^{t}\xi^{T}(t)Q_{1}\xi (t)ds+\int_{t-h-\tau }^{t-\tau }\xi^{T}(t)Q_{2}\xi (t)ds,$$

where P, $Q_{1}$ and $Q_{2}$ are positive definite matrices with the corresponding dimension, respectively. Let:

(52) $$V_{1}(t)=\xi^{T}(t)P\xi (t),$$

(53) $$V_{2}(t)=\int_{t-\tau }^{t}\xi^{T}(t)Q_{1}\xi (t)ds+\int_{t-h-\tau }^{t-\tau }\xi^{T}(t)Q_{2}\xi (t)ds.$$

Then, let k∈{1,2,…,N}, and build the *k*th topology’s Lyapunov function:

(54) $$V^{k}(t)=\left\{\xi^{T}(t)P\xi (t)+\int_{t-\tau }^{t}\xi^{T}(t)Q_{1}\xi (t)ds+\int_{t-h-\tau }^{t-\tau }\xi^{T}(t)Q_{2}\xi (t)ds\right\}1_{\theta (t)=k}.$$

Build the expectation equation E{V(t)} of the Lyapunov function:

(55) $$E\left\{V^{k}(t)\right\}=E\left(\sum_{j=1}^{2}V_{j}\left(t\right)1_{\left\{\theta (t)=k\right\}}\right).$$

The derivation of the Lyapunov function expectation equation is obtained:

(56) $$dE\left\{V^{k}(t)\right\}=E\left(\sum_{j=1}^{2}d\left(V_{j}\left(t\right)1_{\left\{\theta (t)=k\right\}}\right)\right).$$

**Lemma** **1.** *Assume that that* f(t) *is observable, and* $E[f(t)1_{\left\{\theta =i\right\}}]$ *exists, so for any* i∈n*, the following equation holds* [31]:

(57) $$E\left[f(x)d(1_{\left\{\theta =i\right\}})\right]=\sum_{j=1}^{n}\gamma_{ji}E\left[f(x)1_{\left\{\theta =i\right\}}\right]dt+ο(dt).$$

The time delay of communication for UUV formation at any time t≥0, satisfies 0<τ(t)<h, and its derivation satisfies $\dot{\tau }(t)<\kappa <1$, h, κ≥0.

Derive the expectation functions of two Lyapunov functions separately:

(58) $$dE\left\{V_{1}^{k}(t)\right\}=E\left\{d\xi^{T}P\xi +\xi^{T}Pd\xi \right\}1_{\left\{\theta (t)=k\right\}}+E\left\{\xi^{T}P\xi +\xi^{T}P\xi \right\}d1_{\left\{\theta (t)=k\right\}}+o(t),$$

(59) $$\begin{array}{ll} dE\left\{V_{2}^{k}(t)\right\}= & E\left\{\xi^{T}Q_{1}\xi +(1-\kappa )\left(\xi_{\tau }^{T}Q_{2}\xi_{\tau }-\xi_{h\cdot \tau }^{T}Q_{2}\xi_{h\cdot \tau }-\xi_{\tau }^{T}Q_{1}\xi_{\tau }\right)\right\}1_{\left\{\theta (t)=k\right\}}\\ & \;+E\left\{\xi^{T}Q_{1}\xi +(1-\kappa )\left(\xi_{\tau }^{T}Q_{2}\xi_{\tau }-\xi_{h\cdot \tau }^{T}Q_{2}\xi_{h\cdot \tau }-\xi_{\tau }^{T}Q_{1}\xi_{\tau }\right)\right\}d1_{\left\{\theta (t)=k\right\}}+o(t) \end{array}$$

where, in order to simplify the expression of above equations, ξ, $\xi_{\tau }$, and $\xi_{h\cdot \tau }$ represent ξ(t), ξ(t−τ) and ξ(t−h−τ), respectively.

The Lyapunov expectation function of topology set is that $dE\left\{V\right\}=\sum_{k=1}^{N}dE\left\{V^{k}(\cdot )\right\}$. Obviously, the Lyapunov expectation function for the switching topology set can be expressed as follows:

(60) $$dE\left\{V\right\}\leq E\left\{d\xi^{T}P\xi +\xi^{T}Pd\xi \right\}+E\left\{\xi^{T}Q_{1}\xi +(1-\kappa )\left(\xi_{\tau }^{T}Q_{2}\xi_{\tau }-\xi_{h\cdot \tau }^{T}Q_{2}\xi_{h\cdot \tau }-\xi_{\tau }^{T}Q_{1}\xi_{\tau }\right)\right\}.$$

It should be noted that, in Equation (60), all state variables are based on the joint topology set. Therefore, the Laplacian matrices of the joint topology set are $L_{p}^{u}$, $L_{v}^{u}$, $L_{c}^{u}$ and $L_{d}^{u}$.

Substituting the simplified system error state Equation (49) into Equation (60), one obtains:

(61) $$\begin{array}{ll} E\left\{{\dot{V}}_{1}\right\}\leq & E\{\xi^{T}\left(P\;\bar{A}+{\bar{A}}^{T}P\right)\xi -\xi_{\tau }^{T}\left(H_{p}^{T}P+H_{v}^{T}P\right)\xi -\xi^{T}\left(PH_{p}^{}+PH_{v}^{}\right)\xi_{\tau }\\ & \;+\sum_{\theta (t)=1}^{N}\Delta^{T}(t-\tau ){\hat{K}}_{p}^{T}P\xi +\sum_{\theta (t)=1}^{N}\xi^{T}P{\hat{K}}_{p}\Delta (t-\tau )+\sum_{\theta (t)=1}^{N}\Delta^{T}(t-\tau ){\hat{K}}_{v}{}^{T}P\xi_{\tau }\\ & +\sum_{\theta (t)=1}^{N}\xi_{\tau }^{T}P{\hat{K}}_{v}\Delta (t-\tau )+\xi^{T}P\Xi F+F^{T}\Xi^{T}P\xi +\xi^{T}P\hat{W}+{\hat{W}}^{T}P\xi \} \end{array},$$

where $\sum_{\theta (t)=1}^{N}\Delta^{T}(t-\tau ){\hat{K}}_{p}^{T}P\xi +\sum_{\theta (t)=1}^{N}\xi^{T}P{\hat{K}}_{p}\Delta (t-\tau )\leq N\mu_{1}\xi^{T}\mathrm{PP}\xi +\mu_{1}^{-1}\sum_{\theta (t)=1}^{N}\Delta^{T}(t-\tau ){\hat{K}}_{p}^{T}{\hat{K}}_{p}\Delta (t-\tau )$.

In the Lyapunov function, there are $\mu_{1}^{-1}\Delta^{T}(t-\tau ){\hat{K}}_{p}^{T}{\hat{K}}_{p}\Delta (t-\tau )$, so Lemma 2 is deduced to further support the stability analysis of the Lyapunov function in the following.

**Lemma** **2.** *Assuming* $0\leq \delta_{ij}\leq \delta$*,* i,j∈{1,2,…,N}*, one obtains:*

(62) $$\sum_{\theta (t)=1}^{N}\Delta^{T}(t-\tau )\left({\hat{K}}_{p}^{T}{\hat{K}}_{p}\right)\Delta (t-\tau )\;\leq 2\delta^{2}\xi^{T}(t-\tau )\left(\left(L_{p}^{u}+L_{c}^{u}\right)⊗{\bar{K}}_{p}^{T}{\bar{K}}_{p}\right)\xi (t-\tau ).$$

**Proof.**  Since the matrices in Equations (45)–(47) are diagonal matrices, it can be obtained:

(63) $$\Delta (t-\tau )=X_{}^{\theta (t)=k}\eta (t)+Y_{}^{\theta (t)=k}\eta_{l}(t)=\left[\begin{array}{cc} X_{}^{\theta (t)=k} & Y_{}^{\theta (t)=k} \end{array}\right]\left[\begin{array}{c} \eta (t)\\ \eta_{l}(t) \end{array}\right],$$

where the time delay is included in $X_{}^{\theta (t)=k}$ and $Y_{}^{\theta (t)=k}$, and the subscript is used to simplify the expression of the time delay, for example $\Delta_{\tau }=\Delta (t-\tau )$, so:

(64) $$\Delta_{\tau }^{T}{\hat{K}}_{p}^{T}{\hat{K}}_{p}\Delta_{\tau }=\left[\begin{array}{cc} \eta^{T} & \eta_{l}^{T} \end{array}\right]\left[\begin{array}{c} {\left(X_{}^{\theta (t)=k}\right)}^{T}\\ {\left(Y_{}^{\theta (t)=k}\right)}^{T} \end{array}\right]\left({\hat{K}}_{p}^{T}{\hat{K}}_{p}\right)\left[\begin{array}{cc} X_{}^{\theta (t)=k} & Y_{}^{\theta (t)=k} \end{array}\right]\left[\begin{array}{c} \eta \\ \eta_{l} \end{array}\right],$$

Since $X_{}^{\theta (t)=k}$ and $Y_{}^{\theta (t)=k}$ are diagonal matrices, Equation (64) is rewritten to:

(65) $$\Delta_{\tau }^{T}\left({\hat{K}}_{p}^{T}{\hat{K}}_{p}\right)\Delta_{\tau }=\eta^{T}X^{\theta (t)=k}{}^{T}\left({\hat{K}}_{p}^{T}{\hat{K}}_{p}\right)X^{\theta (t)=k}\eta +\eta_{l}^{T}Y^{\theta (t)=k}{}^{T}\left({\hat{K}}_{p}^{T}{\hat{K}}_{p}\right)Y^{\theta (t)=k}\eta_{l},$$

Since ${\hat{K}}_{p}=I_{n}⊗{\bar{K}}_{p}$, and the special structure of ${\bar{K}}_{p}$, the following equation is established:

(66) $$\begin{array}{ll} \Delta_{\tau }^{T}\left({\hat{K}}_{p}^{T}{\hat{K}}_{p}\right)\Delta_{\tau } & =\sum_{i=1}^{n}\eta_{i}^{T}X_{i}^{\theta (t)=k}{}^{T}\left({\bar{K}}_{p}^{T}{\bar{K}}_{p}\right)X_{i}^{\theta (t)=k}\eta_{i}+\sum_{i=1}^{n}\eta_{li}^{T}Y_{i}^{\theta (t)=k}{}^{T}\left({\bar{K}}_{p}^{T}{\bar{K}}_{p}\right)Y_{i}^{\theta (t)=k}\eta_{li}\\ & =\sum_{i=1}^{n}\sum_{j=1}^{n}{\left(a_{ij}^{\theta (t)=k}\right)}^{2}\delta_{ij}^{2}\eta_{{}_{ij}}^{2}{\left(\xi_{j\tau }-\xi_{i\tau }\right)}^{T}\left({\bar{K}}_{p}^{T}{\bar{K}}_{p}\right)\left(\xi_{j\tau }-\xi_{i\tau }\right)+\sum_{i=1}^{n}\eta_{li}^{2}\delta_{li}^{2}c_{li}^{2}\xi_{i\tau }\left({\bar{K}}_{p}^{T}{\bar{K}}_{p}\right)\xi_{i\tau } \end{array},$$

where $X_{i}^{\theta (t)=k}=\left[\begin{array}{cc} X_{pi}^{\theta (t)=k} & \\ & X_{vi}^{\theta (t)=k} \end{array}\right]$, $\eta_{i}(t)=\left[\begin{array}{c} \eta_{i}(t)\\ \eta_{i}(t) \end{array}\right]$, $Y_{i}^{\theta (t)=k}=\left[\begin{array}{cc} Y_{pi}^{\theta (t)=k} & \\ & Y_{vi}^{\theta (t)=k} \end{array}\right]$, $\eta_{li}(t)=\left[\begin{array}{c} \eta_{li}(t)\\ \eta_{li}(t) \end{array}\right]$.

According to the definition of communication links between leader and followers, one obtains:

(67) $$\Delta_{\tau }^{T}\left({\hat{K}}_{p}^{T}{\hat{K}}_{p}\right)\Delta_{\tau }\leq \delta^{2}\sum_{i=1}^{n}\sum_{j=1}^{n}a_{ij}^{\theta (t)=k}{\left(\xi_{j\tau }-\xi_{i\tau }\right)}^{T}\left({\bar{K}}_{p}^{T}{\bar{K}}_{p}\right)\left(\xi_{j\tau }-\xi_{i\tau }\right)+\delta^{2}\sum_{i=1}^{n}c_{ij}^{\theta (t)=k}\xi_{i\tau }\left({\bar{K}}_{p}^{T}{\bar{K}}_{p}\right)\xi_{i\tau }.$$

Equivalent to:

(68) $$\Delta_{\tau }^{T}\left({\hat{K}}_{p}^{T}{\hat{K}}_{p}\right)\Delta_{\tau }\leq 2\delta^{2}\xi_{\tau }^{T}\left(L_{p}^{\theta (t)=k}⊗{\bar{K}}_{p}^{T}{\bar{K}}_{p}\right)\xi_{\tau }+\delta^{2}\xi_{\tau }^{T}\left(L_{c}^{\theta (t)=k}⊗{\bar{K}}_{p}^{T}{\bar{K}}_{p}\right)\xi_{\tau }.$$

Since there must be $\delta^{2}\xi_{\tau }^{T}\left(L_{c}^{\theta (t)=k}⊗{\bar{K}}_{p}^{T}{\bar{K}}_{p}\right)\xi_{\tau }\geq 0$, the following inequality is established:

(69) $$\Delta_{\tau }^{T}\left({\hat{K}}_{p}^{T}{\hat{K}}_{p}\right)\Delta_{\tau }\leq 2\delta^{2}\xi_{\tau }^{T}\left(\left(L_{p}^{\theta (t)=k}+L_{c}^{\theta (t)=k}\right)⊗{\bar{K}}_{p}^{T}{\bar{K}}_{p}\right)\xi_{\tau }.$$

Then, for the joint topological set:

(70) $$\sum_{\theta (t)=1}^{N}\Delta_{\tau }^{T}\left({\hat{K}}_{p}^{T}{\hat{K}}_{p}\right)\Delta_{\tau }\leq 2\delta^{2}\xi_{\tau }^{T}\left(\left(L_{p}^{u}+L_{c}^{u}\right)⊗{\bar{K}}_{p}^{T}{\bar{K}}_{p}\right)\xi_{\tau }.$$

The proof of Lemma 2 is completed. □

In the same way, the following inequality is obtained:

(71) $$\sum_{\theta (t)=1}^{N}\Delta_{\tau }^{T}{\hat{K}}_{v}{}^{T}{\hat{K}}_{v}\Delta_{\tau }\leq 2\delta^{2}\xi_{\tau }^{T}\left(\left(L_{v}^{u}+L_{d}^{u}\right)⊗{\bar{K}}_{p}^{T}{\bar{K}}_{p}\right)\xi_{\tau }.$$

Equation (61) can be rewritten as:

(72) $$\begin{array}{ll} E\left\{{\dot{V}}_{1}\right\}\leq & E\{\xi^{T}\left(P\;\bar{A}+{\bar{A}}^{T}P\right)\xi -\xi_{\tau }^{T}\left(H_{p}^{T}P+H_{v}^{T}P\right)\xi -\xi^{T}\left(PH_{p}^{}+PH_{v}^{}\right)\xi_{\tau }\\ & \;+N\mu_{1}\xi^{T}\mathrm{PP}\xi +2\mu_{1}^{-1}\delta^{2}\xi_{\tau }^{T}\left(\left(L_{p}^{u}+L_{c}^{u}\right)⊗{\bar{K}}_{p}^{T}{\bar{K}}_{p}\right)\xi_{\tau }+\mu_{2}N\xi^{T}\mathrm{PP}\xi \\ & \;+2\mu_{2}^{-1}\delta^{2}\xi_{\tau }^{T}\left(\left(L_{v}^{u}+L_{d}^{u}\right)⊗{\bar{K}}_{p}^{T}{\bar{K}}_{p}\right)\xi_{\tau }+\xi^{T}P\Xi F+F^{T}\Xi^{T}P\xi \\ & \;+\xi^{T}P\hat{W}+{\hat{W}}^{T}P\xi \} \end{array}.$$

Considering the additional nonlinear factors, it is:

(73) $$\xi^{T}P\Xi F+F^{T}\Xi^{T}P\xi \leq \mu_{3}\xi^{T}\mathrm{PP}\xi +\mu_{3}^{-1}F^{T}\Xi^{T}\Xi F\leq \mu_{3}\xi^{T}\mathrm{PP}\xi +\mu_{3}^{-1}\beta p^{2}\xi^{T}\xi .$$

For the disturbances of ocean current:

(74) $$\xi^{T}P\hat{W}+{\hat{W}}^{T}P\xi \leq \mu_{4}\xi^{T}\mathrm{PP}\xi +\mu_{4}^{-1}{\hat{W}}^{T}\hat{W}\leq \mu_{4}\xi^{T}\mathrm{PP}\xi +\varpi^{2}\mu_{4}^{-1}I_{n}^{T}I_{n}.$$

Substituting the Equations (73) and (74) into Equation (72), one obtains:

(75) $$\begin{array}{ll} E\left\{{\dot{V}}_{1}\right\}\leq & E\{\xi^{T}\left(P\;\bar{A}+{\bar{A}}^{T}P\right)\xi -\xi_{\tau }^{T}\left(H_{p}^{T}P+H_{v}^{T}P\right)\xi -\xi^{T}\left(PH_{p}+PH_{v}\right)\xi_{\tau }\\ & +N\mu_{1}\xi^{T}\mathrm{PP}\xi +2\mu_{1}^{-1}\delta^{2}\xi_{\tau }^{T}\left(\left(L_{p}^{u}+L_{c}^{u}\right)⊗{\bar{K}}_{p}^{T}{\bar{K}}_{p}\right)\xi_{\tau }+\mu_{2}N\xi^{T}\mathrm{PP}\xi \\ & +2\mu_{2}^{-1}\delta^{2}\xi_{\tau }^{T}\left(\left(L_{v}^{u}+L_{d}^{u}\right)⊗{\bar{K}}_{p}^{T}{\bar{K}}_{p}\right)\xi_{\tau }+\mu_{3}\xi^{T}\mathrm{PP}\xi +\mu_{3}^{-1}\beta p^{2}\xi^{T}\xi \\ & +\mu_{4}\xi^{T}\mathrm{PP}\xi +\varpi^{2}\mu_{4}^{-1}I_{n}^{T}I_{n}\} \end{array}.$$

Combining the Equations (58), (59) and (75), the following is obtained:

(76) $$\begin{array}{ll} E\{\dot{V}\}\leq & E\{\xi^{T}\left(P\;\bar{A}+{\bar{A}}^{T}P\right)\xi -\xi_{\tau }^{T}\left(H_{p}^{T}+H_{v}^{T}\right)P\xi -\xi^{T}P\left(H_{p}+H_{v}\right)\xi_{\tau }\\ & \;+N\mu_{1}\xi^{T}\mathrm{PP}\xi +2\mu_{1}^{-1}\delta^{2}\xi_{\tau }^{T}\left(\left(L_{p}^{u}+L_{c}^{u}\right)⊗{\bar{K}}_{p}^{T}{\bar{K}}_{p}\right)\xi_{\tau }+\mu_{2}N\xi^{T}\mathrm{PP}\xi \\ & \;+2\mu_{2}^{-1}\delta^{2}\xi_{\tau }^{T}\left(\left(L_{v}^{u}+L_{d}^{u}\right)⊗{\bar{K}}_{p}^{T}{\bar{K}}_{p}\right)\xi_{\tau }+\mu_{3}\xi^{T}\mathrm{PP}\xi +\mu_{3}^{-1}\beta p^{2}\xi^{T}\xi \\ & \;+\mu_{4}\xi^{T}\mathrm{PP}\xi +\varpi^{2}\mu_{4}^{-1}\gamma^{2}\xi^{T}\xi +\xi^{T}Q_{1}\xi +(1-\kappa )\xi_{\tau }^{T}\left(Q_{2}-Q_{1}\right)\xi_{\tau }-(1-\kappa )\xi_{h\cdot \tau }^{T}Q_{2}\xi_{h\cdot \tau }\} \end{array},$$

If Equation (50) holds, there must be a positive real number σ>0, so that $E\{\dot{V}\}\leq -\sigma {\left\|\xi (t)\right\|}^{2}$. Therefore, the leader-follower UUV formation control is asymptotically stable.

## 5. Simulations

**Figure 4:**
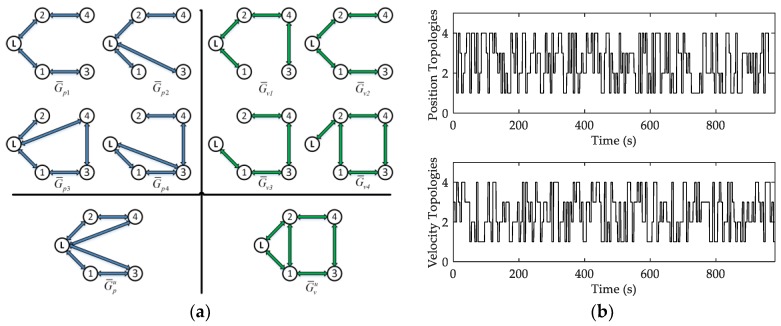
Switching topologies: (**a**) the two-layer random switching topology set; and (**b**) the Markov random states in switching topology.

To illustrate the theoretical results obtained in the previous sections, some simulations are given. Suppose that the leader-follower UUV formation is consisted of one leader and four followers. The time delay is τ(t)=0.1(1+sin(2t)). The two-layer random switching topology set contain four (N=4) position communication topologies ${\bar{G}}_{pi}$ and four velocity communication topologies ${\bar{G}}_{vi}$, i∈{1,2,3,4}. The joint topologies are ${\bar{G}}_{p}^{u}$ and ${\bar{G}}_{v}^{u}$ which are shown in Figure 4a. Figure 4b shows the state changes of the two-layer Markov switching topology in this simulation example.

The formation structure is designed as an equilateral triangle in this simulation, and each follower maintains a desired geometric configuration with the leader. The follower UUVs include UUV 1, UUV 2, UUV 3, and UUV 4. The desired relative distance $\Delta l_{1}^{d}$ of UUV 1 to the leader is 10 m, the desired relative distance $\Delta l_{2}^{d}$ of UUV 2 is 10 m, the desired relative distance $\Delta l_{3}^{d}$ of UUV 3 is 20 m, and the desired relative distance $\Delta l_{4}^{d}$ of UUV 4 is 20 m. Relative angle $\varphi_{i}^{}$ is the angle between the motion direction of leader and relative distance. The desired relative angle $\varphi_{1}^{d}$ of UUV 1 is 150 deg, desired relative angle $\varphi_{2}^{d}$ of UUV 2 is 210 deg, desired relative angle $\varphi_{3}^{d}$ of UUV 3 is 150 deg, and desired relative angle $\varphi_{4}^{d}$ of UUV 4 is 210 deg.

The initial position of each follower is randomly placed in the three-dimensional space. The initial pitch angle and heading angle are respectively set in the range [−π/18,π/18] and [0,2π]. The initial states of leader UUV and all follower UUVs are shown in Table 1. 

The control gains of the leader-follower UUV formation controller are $K_{p}=k_{\alpha }{\left[\begin{array}{cc} 0 & I \end{array}\right]}^{T}\in {\mathbb{R}}^{5\times 10}$ and $K_{v}=k_{\beta }{\left[\begin{array}{cc} 0 & I \end{array}\right]}^{T}\in {\mathbb{R}}^{5\times 10}$, where $k_{\alpha }=0.045$, $k_{\beta }=0.58$. The leader is operated on the desired path which is designed as a spiral curve:

(77) $$\left\{ \begin{array}{l} x(t)=100\cos(0.02\pi t)\\ y(t)=100\sin(0.02\pi t)\\ z(t)=-0.03t-2 \end{array}\right..$$

Current velocity is set as $U_{B}=[0.2,\;0.2,\;0]\;m/s$. The SNR of the acoustic communication is selected as 10 dB, and all effective communication topology weights are considered under the condition of SNR 10 dB. The additional nonlinear function is defined as the saturation function related to the UUV’s velocity state as shown in Equation (78) and satisfies the Poisson distribution: $P(f(t,x_{i}))=0.5$:

(78) $$f(t,x_{i})=0.01\tanh(v_{i}(t)).$$

**Figure 5:**
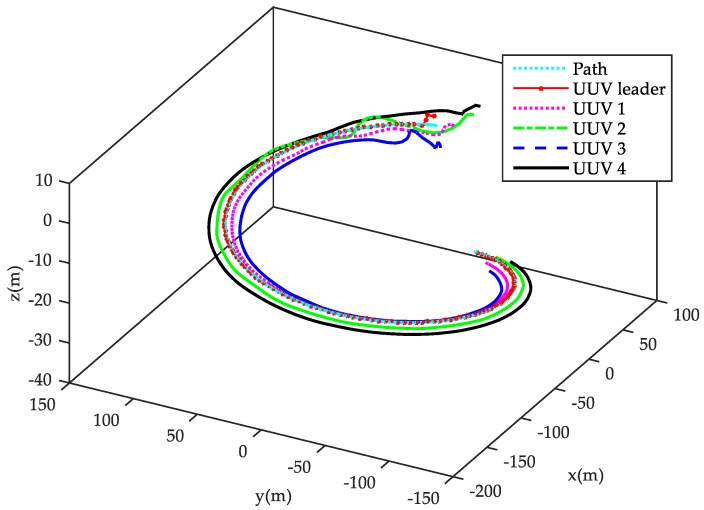
3D trajectory of the leader-follower UUV formation.

**Figure 6:**
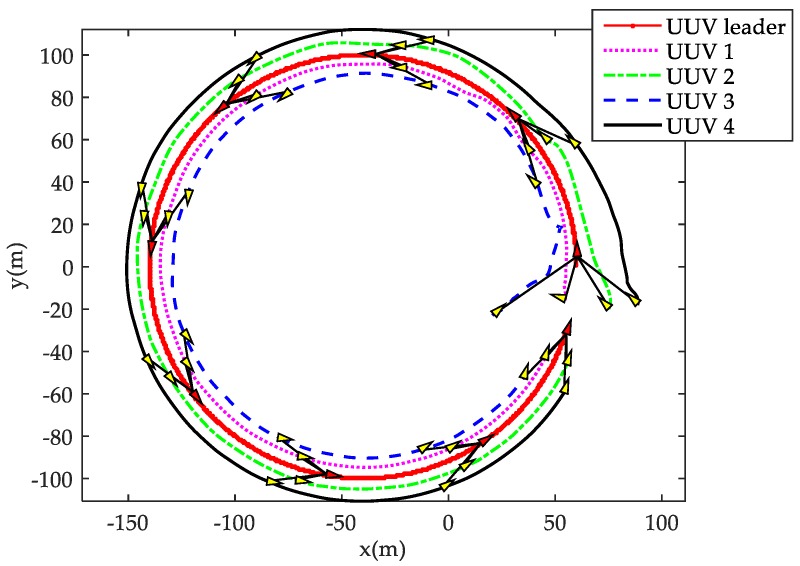
The trajectory of leader and follower UUVs in 2D with the desired triangle formation structure.

**Figure 7:**
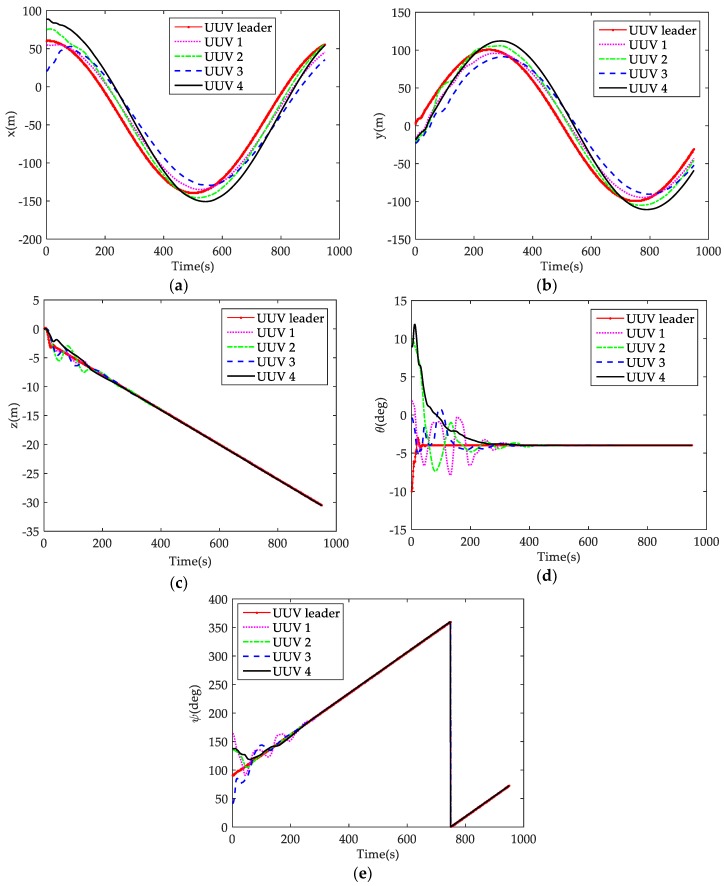
Position and attitude states of the UUVs: (**a**) state x of each UUV; (**b**) state y of each UUV; (**c**) state z of each UUV; (**d**) pitch θ of each UUV; and (**e**) heading ψ of each UUV.

**Figure 8:**
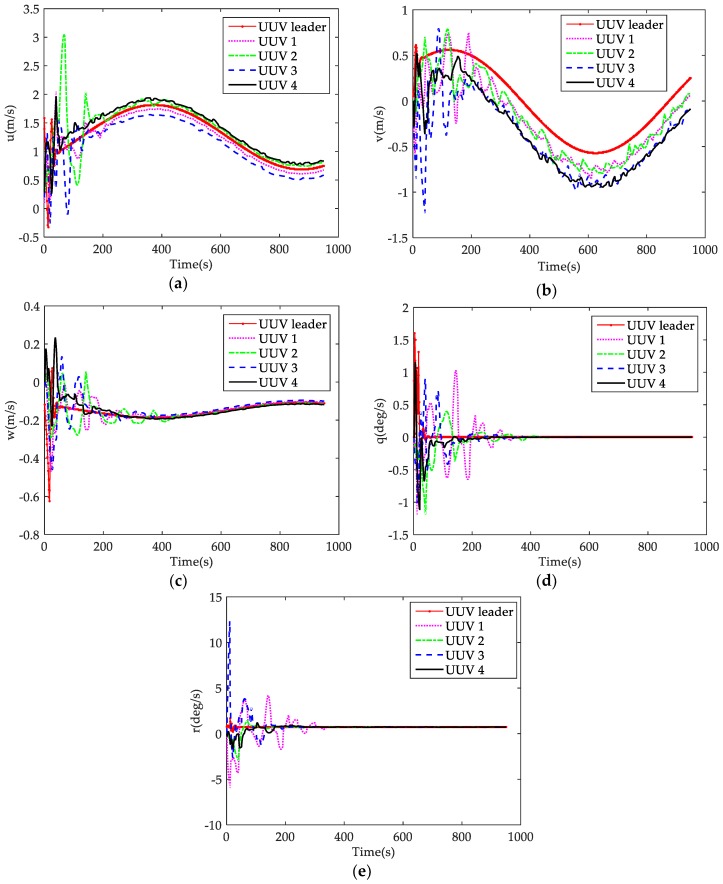
Velocity states of the UUVs: (**a**) velocity u of each UUV; (**b**) velocity v of each UUV; (**c**) velocity w of each UUV; (**d**) angular velocity q of each UUV; and (**e**) angular velocity r of each UUV.

Figure 5, Figure 6, Figure 7 and Figure 8 show the simulation results of the leader-follower UUV formation control.

Figure 5 shows the three-dimensional trajectory of each member in the leader-follower UUV formation. As shown in the figure, the leader UUV is responsible for tracking the desired spiral curve path, and each follower UUV tracks the leader UUV according to its desired relative distance and angle, regardless of tracking the spiral curve path. The ultimate control result is that the whole multi-UUV system tracks the desired spiral path with the desired triangle formation structure. Figure 5 shows that, after an adjustment period, the randomly-placed follower UUVs can stably converge to a desired formation structure, and the leader-follower formation can, primarily, keep tracking the desired path. The two-dimensional trajectory of the leader and follower UUVs are shown in Figure 6. The red and yellow filled triangles on each trajectory denote the heading of the leader UUV and follower UUVs, respectively. It can be shown in Figure 6 that the initial positions and headings of each follow UUV are disorderly, which is not conducive to constructing a formation and track spatial path. However, under the formation control law, each follower can reach and keep the desired relative distance and relative angle with the leader after an adjustment period, and the leader and all followers can maintain the desired triangle formation structure.

Figure 7 shows the position and attitude states of leader-follower UUV formation. It can be seen detailed from the x, y, and z position figure, and the desired triangle formation absolutely follows the desired path after about 170 s because of the initial random positions of the follower UUVs. For Figure 7a, by analyzing the response trend of the position x value for all UUVs, it can be found that the leader UUV gradually shifts to be the smallest and then gradually shifts to be the largest. Referring to Figure 6, the response trend of the position x value is totally reasonable according to the position relationships of all UUVs along the two-dimensional circle trajectory. The similar analysis can be conducted regarding the response trend of the position y value in Figure 7b. From the pitch and heading figure, it can be shown that the attitudes of the UUVs present a period of adjustments because of model uncertainties and current disturbances. However, the pitch and heading of all follower UUVs finally converge to the leader UUV.

Figure 8 shows the velocity states of the leader-follower UUV formation. As shown in the figure, UUVs make a large adjustment of velocities early, and also a small adjustment when the followers converge to the desired formation structure after 300 s. This is because of changes of the transformation topology and the nonlinear function due to speed changes. It can also be observed that the follower UUVs located outside the desired spiral path (UUV 2 and UUV 4) have a greater surge velocity u than the follower UUVs located inside the path (UUV 1 and UUV 3), which can verify the correctness and effectiveness of the formation control algorithm. By reason of the desired helix path, it can be found that velocity v of all follower UUVs are mainly adjusted when the formation is maintained. The angular velocity q and r of all follower UUVs make adjustments before about 300 s, and also finally converge to the leader UUV.

## 6. Discussion

As is known, the leader-follower approach is a main formation control method of UUVs, and its basic principles and algorithms are relatively mature. In recent years, in order to obtain better application, the studies have focused on more practical problems when adopting the leader-follower approach. These problems mainly involve three aspects. One is the self-problem of UUVs for nonlinearity, under-actuation, control input saturation, and time-varying parameters. Another is the environment disturbance problem of ocean currents, waves, and obstacles. The last is the communication problem of delay, failure, and link noise interference. 

To the best of our knowledge, most studies cover only one or two of the three aspects. Especially for communication problems, fewer studies involve communication failure and link noise interference, which have a seriously impact on the stability of formation control. However, for the purpose of developing a practical and effective formation control method, the three aspects are simultaneously considered in this paper. Moreover, in order to model and solve the problems, some novel means and ways are adopted. The main originalities of the paper can be summarized as follows:

First, three problems of model uncertainties, current disturbances, and unstable communication are simultaneously considered and modeled. Based on the three models, leader-follower formation controller is designed in the paper. More importantly, the stability and convergence condition of the controller is proposed and proved using the Lyapunov-Razumikhin theorem. Second, for model uncertainties, time-varying parameters and the nonlinearity of the UUV are modeled as a bounded nonlinear function. The nonlinear function exists as a certain probability meeting Bernoulli’s distribution, which is more in line with the actual situation. Third, communication failure and acoustic link noise interference are both modeled and solved by the method of converting to different communication topology problems. The communication failure problem is modeled and converted to a two-layer random switching communication topology. The acoustic noise interference problem is modeled and represented by effective communication topology weights.

As mentioned in Section 2.1, the method and algorithm proposed also have application boundary conditions in mission scenarios, system scale, communication frequency, formation spacing, and so on. Further, in order to improve the method and make it a more practical implementation, the two following future research directions may need to be concerned. One research direction is to develop formation control algorithms based on limited state information of the leader, which can reduce the communication burden and communication delay. The other one is to add effective estimate algorithms for position and velocity states of both the leader and all follower UUVs to tolerate the unstable communication.

## 7. Conclusions

This paper addressed the problems of leader-follower UUV formation control with model uncertainties, current disturbances, and unstable communication. A second-order integral UUV model with nonlinear function and current disturbances is established by state feedback linearization. Thus, the model of the UUV is divided into an approximate linear part and a nonlinear uncertain part. Unstable communication considered in this paper includes communication failure and acoustic link noise interference. Then, two-layer random switching topologies are designed, which can dynamically switch to solve the problem of communication failure. The concept of communication topology effective weight is proposed to represent the validity of communication information interfered by noises in acoustic link. By stripping noise disturbances, utilizing a state feedback and consensus algorithm, the distributed controllers of all follower UUVs are obtained. Using the Lyapunov-Razumikhin theorem, the asymptotic stability of the leader-follower UUV formation control method designed in this paper is proven. The effectiveness of the method is simulated by tracking a spiral helix curve path with one leader UUV and four follower UUVs. The simulation results show that leader-follower UUV formation controllers are feasible and effective. After a period of adjustment, all follower UUVs can converge to a desired formation structure, and the formation can keep tracking the desired path.

## Figures and Tables

**Table 1:** The initial states of the leader UUV and all follower UUVs.

	Initx (m)	Inity (m)	Initz (m)	Initθ (deg)	Initψ (deg)	Initu (m/s)	Initv (m/s)	Initw (m/s)
Leader	60	3	0	–10	90	1.58	0.1	–0.12
UUV 1	54.5	–15.6	0	1.83	164	0.1	0	0
UUV 2	75.1	–20	0	10	135	0.27	0	0
UUV 3	20.6	–23	0	0	41.8	0.2	0	0
UUV 4	88.6	–18	0	8.9	137.6	0.28	0	0

## Appendix A

**Table A1:** List of main symbols.

Symbol	Description
x, y, z	Position in surge, sway, heave
θ, ψ	Pitch, yaw
u, v, w	Linear velocity in surge, sway, heave
q, r	Pitch velocity, yaw velocity
$x={\left[x,y,z,\theta ,\psi \right]}^{T}$	Position and orientation vector of UUV
$v={\left[u,v,w,q,r\right]}^{T}$	Linear and angular velocity vector of UUV
W	Gravity
B	Buoyancy
$r_{B}^{W}=[0,0,0]$	The coordinates of gravity center in body-fixed frame
$r_{B}^{B}=[x_{B}^{B},y_{B}^{B},z_{B}^{B}]$	The coordinates of buoyancy center in body-fixed frame
$X_{prop},Y_{prop},Z_{prop}$	Thrusts
$\delta_{r},\delta_{s}$	Steering angles
m	Weight of the UUV
n	Total number of follower uuvs
$x_{i}^{}(t)$	The *i*th follower’s position and orientation vector, i=1,2,…n
$v_{i}^{}(t)$	The *i*th follower’s velocity vector, i=1,2,…n
$\varepsilon_{i}(t)={\left[\begin{array}{cc} x_{i}^{T}(t) & v_{i}^{T}(t) \end{array}\right]}^{T}$	The *i*th follower’s state vector, i=1,2,…n
$x_{l}^{}(t)$	The leader’s position and orientation vector
$v_{l}^{}(t)$	The leader’s velocity vector
$\varepsilon_{l}(t)={\left[\begin{array}{cc} x_{l}^{T}(t) & v_{l}^{T}(t) \end{array}\right]}^{T}$	The leader’s state vector
$\varepsilon (t)={\left[\begin{array}{cccc} \varepsilon_{1}^{T}(t) & \varepsilon_{2}^{T}(t) & \cdots & \varepsilon_{n}^{T}(t) \end{array}\right]}^{T}$	State vectors of all followers
$\xi_{i}(t)=\varepsilon_{i}(t)-\varepsilon_{l}(t)$	The *i*th follower’s state error vector
$\xi (t)={\left[\xi_{1}^{T}(t),\ldots ,\xi_{n}^{T}(t)\right]}^{T}$	The formation system’s state error vector
$U_{E}=(u_{e},v_{e},w_{e})$	Ocean current velocities in earth reference frame
$U_{B}=(u_{b},v_{b},w_{b})$	Ocean current velocities in body-fixed frame
ϑ(t)	Bernoulli’s distribution function
N	The quantity of the topological set
${\bar{G}}_{pi}({\bar{V}}_{pi},{\bar{\varepsilon }}_{pi},{\bar{A}}_{pi})$	The *i*th position topological unit in position topology set, i=1,2,…N
${\bar{G}}_{vi}({\bar{V}}_{vi},{\bar{\varepsilon }}_{vi},{\bar{A}}_{vi})$	The *i*th velocity topological unit in velocity topology set, i=1,2,…N
${\bar{G}}_{p}^{u}≜{\bar{G}}_{p1}\cup \ldots \cup {\bar{G}}_{pN}$	Joint topology of position topology set
${\bar{G}}_{v}^{u}≜{\bar{G}}_{v1}\cup \ldots \cup {\bar{G}}_{vN}$	Joint topology of velocity topology set
$\gamma_{ij}$	The switching probability from topology i to topology j, i,j=1,2,…N
$K_{p}$, $K_{v}$	Control gains for the position and velocity communication topologies
